# The relationship between sexual self-concept and sexual function among women who undergone hysterectomy: a cross-sectional study in western Iran

**DOI:** 10.1515/med-2026-1486

**Published:** 2026-07-22

**Authors:** Zahra Zamiri Araste, Salman Khazaei, Zahra Karami, Ensiyeh Jenabi

**Affiliations:** Department of Midwifery, School of Nursing and Midwifery, Hamadan University of Medical Sciences, Hamadan, Iran; Research Center for Health Sciences, Institute of Health Sciences and Technology, Hamadan University of Medical Sciences, Hamadan, Iran; Student Research Committee, Hamadan University of Medical Sciences, Hamadan, Iran; Mother and Child Care Research Center, Institute of Health Sciences and Technology, Hamadan University of Medical Sciences, Hamadan, Iran

**Keywords:** sexual function, sexual self-concept, hysterectomy, Iran

## Abstract

**Objectives:**

Hysterectomy is one of the most significant gynecological surgeries and can profoundly affect various aspects of women’s health. This study aimed to investigate the relationship between sexual self-concept and sexual function in women who have undergone hysterectomy in Hamadan City, western Iran.

**Methods:**

This cross-sectional analytical study was conducted from June 2024 to June 2025 at Fatemieh Hospital in Hamadan. A total of 200 women who have undergone hysterectomy were enrolled using convenience sampling. Demographic and obstetric questionnaire, the Female Sexual Function Index (FSFI), and the Multidimensional Sexual Self-Concept Questionnaire (MSSCQ) were completed for all participants. Data analysis was performed using Stata software version 17.

**Results:**

In this study, 200 participants were evaluated. The mean age of the participants was 47.84 years (SD=6.75). Sexual function scores were significantly higher in women with higher education levels (p<0.001), employed women (p=0.022), those with higher spousal education levels (p<0.001), those with employed spouses (p<0.001), and across different ethnic backgrounds (p=0.029). Additionally, a strong positive correlation was observed between the total FSFI and total MSSCQ scores (r=0.93, p<0.001).

**Conclusions:**

The present study revealed a significant positive correlation between total sexual self-concept scores and sexual function. Preliminary evidence for future longitudinal or interventional sexual research can provide a basis for intervention strategies.

## Introduction

Hysterectomy is one of the most common major surgical procedures worldwide [1]. Overall incidence of hysterectomy was 1.1 per 1,000 births [2]. Although the rate in Iran is also high and increasing, accurate national data on hysterectomy prevalence remain unavailable [3]. As one of the most significant gynecological surgeries, hysterectomy targets the uterus, an organ often integral to feminine identity, and can profoundly affect various aspects of women’s health, including quality of life, mental health, and sexual well-being [4]. The loss of female reproductive organs, as occurs in hysterectomy, whether visibly apparent or not, leads to profound changes in attitudes and sexual self-concept [5].

Sexual self-concept is a fundamental component of sexual health, encompassing an individual’s positive and negative perceptions of themselves as a sexual being [6]. It appears to play a guiding role in sexual functioning [4]. This multidimensional construct includes: Positive aspects (sexual self-efficacy, sexual awareness, sexual optimism, avoidance of risky sexual behaviors, self-blame in cases of sexual difficulties, and sexual management); negative aspects (sexual anxiety, monitoring of sexual desires, fear of sexual relationships, and sexual depression) and situational aspects (sexual preoccupation, sexual assertiveness, sexual motivation, and sexual self-schema) [7]. As one of the most significant predictors and guiding factors of sexual function, sexual self-concept profoundly influences how individuals experience and engage in sexual behaviors [6].

Physiological responses that occur following sexual stimulation are referred to as sexual response and function [8]. The effects of hysterectomy on sexual function vary among individuals. Common complaints following hysterectomy include: loss of sexual desire, reduced frequency of intercourse, diminished sexual response, difficulty achieving orgasm, sensation of vaginal tightness, dyspareunia, vaginal shortening, loss of penile penetration depth and reduced vaginal lubrication and elasticity [9].

The study by Riyazi et al. reported that sexual motivation and sexual satisfaction play important roles in sexual function among infertile women [10]. Other study conducted by Dedden et al. showed that hysterectomy is not associated with significant changes in sexual function, and hysterectomy with ovarian preservation may even be linked to improvements in certain domains of sexual function, such as lubrication and orgasm [4]. Given the lack of existing studies investigating the relationship between sexual self-concept and sexual function in women who have undergone hysterectomy, this study aims to address this gap by examining the association between sexual self-concept and sexual function among women who have undergone hysterectomy in western Iran.

## Materials and methods

### Participants

This cross-sectional study was conducted on 200 women who undergone hysterectomy between June 2024 and June 2025 for duration of one year at the Fatemieh Hospital, a referral hospital located in Hamadan province, western Iran.

### Ethical

The research protocol was reviewed and approved by the Ethics Committee of Hamadan University of Medical Sciences (approval code: IR.UMSHA.REC.1403.353), in accordance with national and international ethical standards for research involving human participants. Written informed consent was obtained from all participants prior to their inclusion in the study.

### Inclusion and exclusion criteria

Eligible participants were women aged 40–65 years who had undergone a total abdominal hysterectomy without oophorectomy for benign indications. Exclusion criteria included a history of infertility; physical health problems in either partner; diagnosed psychiatric disorders in either partner; experience of a stressful life event within the past three months in either partner; alcohol or substance abuse in either partner; sexual dysfunction under treatment in either partner; and the use of medications known to affect sexual desire in either partner, such as antidepressants, antihistamines, narcotics, spironolactone, antihypertensive agents, and anticonvulsants.

### Sample size

According to the study by Jafarpoor et al. [11], which reported a correlation of 0.23 between positive sexual self-concept and sexual function in married women (p<0.001, r=0.23), and considering a test power of 90 % and a type I error rate of 0.05, the required sample size was estimated to be 200 participants.

### Sampling

Participants were recruited using a convenience sampling method until the predetermined sample size was achieved. One of the researchers (Z.Z.) approached women who had undergone hysterectomy at Fatemieh Hospital. After initial selection, eligibility was confirmed based on the inclusion and exclusion criteria. The study objectives were explained in detail, and women who provided informed consent subsequently completed the questionnaires.

### Measurement tools

The tools were including the demographic characteristics, the multidimensional sexual self-concept questionnaire (MSSCQ) and Female Sexual Function Index (FSFI) Questionnaire.

### Demographic and obstetrics questionnaire

The demographic and obstetrics questionnaire addressed personal characteristics, including variables such as age, spouse’s age, duration of marriage, occupation, spouse’s occupation, educational level, spouse’s educational level, ethnicity, religion, economic status, number of pregnancies, number of children, availability of a private room at home, and frequency of intercourse per month.

### The multidimensional sexual self-concept questionnaire (MSSCQ)

Snell et al. developed the MSSCQ, the questionnaire comprises 100 items across 20 dimensions, with five items per dimension, assessing cognitive (sexual self-schemata), emotional (sexual depression), and motivational (sexual motivation) aspects [12]. Respondents rate each item, usually on a Likert scale, allowing researchers to measure both positive and negative dimensions of sexual self-concept. Higher scores typically indicate stronger or more positive sexual self-concept in the corresponding domain. The MSSCQ has been widely adapted and validated in different cultures, including a Farsi version for Iranian populations. The Persian version of the MSSCQ consists of 78 items covering 18 domains of an individual’s sexual life. The items are scored on a 5-point Likert scale, with higher scores indicating a greater sexual self-concept in each domain. In the present study, sexual self-concept was examined across three overarching dimensions: positive, negative, and situational. The Farsi version of the MSSCQ has demonstrated good psychometric properties in Iranian populations. Specifically, studies have shown acceptable to good reliability (including Cronbach’s alpha scores above 0.80) and evidence of both convergent and discriminant validity [13].

### Female Sexual Function Index (FSFI) Questionnaire

The Female Sexual Function Index (FSFI) is a self-administered questionnaire comprising 19 items that evaluate multiple dimensions of female sexual functioning over the preceding month. It assesses six domains: desire (2 items), subjective arousal (4 items), lubrication (4 items), orgasm (3 items), satisfaction (3 items), and pain (3 items). Total scores range from 2 to 36, with higher scores reflecting better sexual function, while lower scores indicate no sexual activity during the past month. The cutoff point for determining sexual dysfunction is a score of 28 or less [14], 15]. The FSFI has been validated in Iran by Babakhanian et al. with a Cronbach’s alpha exceeding 0.80 for both the entire scale and its subscales [16].

### Statistical analysis

Descriptive statistics, including mean and standard deviation for quantitative variables and frequency and percentage for qualitative variables, were used to describe the study population. The mean scores of women’s positive sexual self-concept and their sexual function across demographic variables were compared using t-test and ANOVA. The correlation between positive sexual self-concept and sexual function in married women was assessed using Pearson’s correlation coefficient. Data were analyzed using Stata software, version 17, with a significance level set at 0.05.

## Results

Table 1 presents the descriptive statistics of continuous variables among 200 women with hysterectomy. The mean age of participants was 47.84 years (SD=6.75) and that of their husbands 55.88 years (SD=7.45), with an average marriage duration of 31.37 years (SD=6.85). Women reported on average 3.11 pregnancies (SD=1.26) and 2.77 children (SD=1.16), and the mean monthly income was 16.52 million Rials (SD=5.80).

**Table 1:** Descriptive statistics of continuous variables among women with hysterectomy.

Variable	Mean	SD	Min	Max
Age, years	47.84	6.75	28	79
Husband’s age, years	55.88	7.45	34	85
Marriage duration, years	31.37	6.85	8	56
Number of pregnancies	3.11	1.26	1	9
Number of children	2.77	1.16	1	8
Monthly income (million rials)	16.52	5.80	6.5	31

Table 2 presents the distribution of categorical socio-demographic characteristics. Regarding education, most participants had secondary (32.00 %) or primary (27.00 %) education, while 79.00 % were unemployed. Their husbands were predominantly employed (89.00 %) and had secondary education (36.50 %) most frequently. The main ethnic groups were Turk (29.00 %) and Fars (28.00 %), and the majority was Shia (83.00 %). More than half lived in personal housing (63.50 %), 48.00 % reported 4–8 sexual intercourses per month, and only 8.50 % had consanguinity marriage, while 68.50 % had a separate bedroom.

**Table 2:** Distribution of categorical socio-demographic characteristics among women with hysterectomy.

Variable	Category	n	%
Education	Primary	54	27.0
	Secondary	64	32.0
	High school	38	19.0
	Diploma	26	13.0
	University	18	9.0
Job	Employed	42	21.0
	Unemployed	158	79.0
Husband’s education	Primary	32	16.0
	Secondary	73	36.5
	High school	45	22.5
	Diploma	28	14.0
	University	22	11.0
Husband’s job	Employed	178	89.0
	Unemployed	22	11.0
Ethnicity	Turk	58	29.0
	Kurd	41	20.5
	Lor	45	22.5
	Fars	56	28.0
Religion	Shia	166	83.0
	Sunni	34	17.0
Housing status	Personal	127	63.5
	Rental	73	36.5
Frequency of intercourse per month	<4 times	54	27.0
	4–8 times	96	48.0
	8–12 times	42	21.0
	>12 times	8	4.0
Consanguinity marriage Separate bedroom	Yes	17	8.5
	No	183	91.5
	Yes	137	68.5
	No	63	31.5

The mean total Female Sexual Function Index (FSFI) score was 22.16 (SD=4.85), and the mean total Multidimensional Sexual Self-Concept Questionnaire (MSSCQ) score was 225.33 (SD=43.36).

Table 3 presents the results of one-way ANOVA assessing the association between socio-demographic variables and total FSFI scores among women with hysterectomy. Sexual function scores were significantly higher in women with higher education (p<0.001) and in those employed (p=0.022). Similarly, higher levels of husband’s education (p<0.001) and husband’s employment (p<0.001) were associated with better FSFI scores. Ethnic differences were also significant (p=0.029), while religion (p=0.089) and housing status (p=0.497) were not related to sexual function. Frequency of monthly intercourse showed a strong positive association with FSFI scores (p<0.001). No significant difference was observed with consanguinity marriage (p=0.884), whereas having a separate bedroom (p<0.001) was significantly associated with higher sexual function.

**Table 3:** Association between socio-demographic factors and total FSFI score among women with hysterectomy.

Variable	Category	Mean ± SD	F (df)	p-Value
Education	Primary	19.47 ± 5.52	F(4,195)=8.20	<0.001
	Secondary	22.97 ± 3.87		
	High school	23.36 ± 3.88		
	Diploma	24.09 ± 3.24		
	University	23.31 ± 3.61		
Job	Employed	23.71 ± 3.85	F(1,198)=5.29	0.022
	Unemployed	21.89 ± 4.71		
Husband’s education	Primary	19.65 ± 5.90	F(4,195)=5.32	<0.001
	Secondary	21.75 ± 4.65		
	High school	23.24 ± 3.80		
	Diploma	23.48 ± 3.49		
	University	24.30 ± 3.01		
Husband’s job	Employed	23.05 ± 3.86	F(1,198)=59.97	<0.001
	Unemployed	15.99 ± 5.31		
Ethnicity	Turk	22.64 ± 4.66	F(3,196)=3.07	0.029
	Kurd	21.27 ± 5.15		
	Lor	23.76 ± 3.19		
	Fars	21.44 ± 4.81		
Religion	Shia	22.52 ± 4.41	F(1,198)=2.92	0.089
	Sunni	21.05 ± 5.34		
Housing status	Personal	22.44 ± 4.57	F(1,198)=0.46	0.497
	Rental	21.98 ± 4.66		
Frequency of intercourse per month	<4 times	19.67 ± 5.72	F(3,196)=9.63	<0.001
	4–8 times	23.14 ± 3.88		
	8–12 times	23.78 ± 3.06		
	>12 times	21.51 ± 4.06		
Consanguinity marriage	Yes	22.12 ± 5.33	F(1,198)=0.02	0.884
	No	22.29 ± 4.54		
Separate bedroom	Yes	23.11 ± 3.89	F(1,198)=15.42	<0.001
	No	20.46 ± 5.45		

Table 4 shows the relationship between socio-demographic factors and total MSSCQ scores among women with hysterectomy. Significant differences were found based on women’s education (p<0.001), husband’s education (p=0.009), women’s employment status (p=0.05), husband’s employment status (p<0.001), ethnicity (p=0.006), frequency of monthly sexual intercourse (p<0.001), and having a private bedroom (p=0.0015). Women with higher education and employment had higher MSSCQ scores. Similarly, women whose husbands were employed scored higher. Lor ethnicity and moderate frequency of sexual intercourse (8–12 times per month) were associated with better scores. No significant differences were seen for religion (p=0.075), housing status (p=0.35), or consanguinity marriage (p=0.751).

**Table 4:** Association between socio-demographic factors and total MSSCQ score among women with hysterectomy.

Variable	Category	Mean ± SD	F (df)	p-Value
Education	Primary	212.67 ± 58.71	F(4,195)=6.39	<0.001
	Secondary	243.98 ± 39.32		
	High school	249.97 ± 40.82		
	Diploma	256.08 ± 33.41		
	University	238.72 ± 33.20		
Job	Employed	250.36 ± 38.89	F(1,198)=3.87	0.05
	Unemployed	234.42 ± 48.47		
Husband’s education	Primary	216.19 ± 60.90	F(4,195)=3.49	0.009
	Secondary	232.86 ± 48.46		
	High school	245.40 ± 40.94		
	Diploma	253.50 ± 32.70		
	University	249.77 ± 33.27		
Husband’s job	Employed	245.23 ± 39.38	F(1,198)=51.15	<0.001
	Unemployed	177.36 ± 59.60		
Ethnicity	Turk	237.60 ± 48.78	F(3,196)=4.30	0.006
	Kurd	226.10 ± 51.05		
	Lor	257.98 ± 30.69		
	Fars	230.23 ± 48.77		
Religion	Shia	240.45 ± 45.23	F(1,198)=3.21	0.075
	Sunni	224.68 ± 53.55		
Housing status	Personal	240.11 ± 45.98	F(1,198)=0.87	0.35
	Rental	233.68 ± 48.74		
Frequency of intercourse per month	<4 times	214.98 ± 59.22	F(3,196)=7.57	<0.001
	4–8 times	244.19 ± 40.92		
	8–12 times	254.86 ± 31.84		
	>12 times	224.75 ± 32.11		
Consanguinity marriage	Yes	241.24 ± 47.94	F(1,198)=0.10	0.751
	No	237.44 ± 47.02		
Private bedroom	Yes	244.85 ± 40.48	F(1,198)=10.34	0.0015
	No	222.37 ± 56.04		

As shown in Table 5, all sub-domains of sexual self-concept except sexual assertiveness (p=0.167) and sexual optimism (p=0.079) showed statistically significant positive correlations with total FSFI scores (p<0.05). The strongest correlations were observed for sexual risk avoidance (r=0.846, p<0.001), sexual self-efficacy (r=0.840, p<0.001), sexual schemas (r=0.840, p<0.001), and sexual motivation (r=0.828, p<0.001).

**Table 5:** Correlation between MSSCQ sub-domains and total FSFI score.

MSSCQ sub-domain	Correlation coefficient (r)	p-Value
Sexual risk avoidance	0.846	<0.001
Sexual self-efficacy	0.840	<0.001
Sexual schemas	0.840	<0.001
Sexual motivation	0.828	<0.001
Sexual self-blame	0.814	<0.001
Sexual prevention	0.813	<0.001
Sexual depression	0.804	<0.001
Sexual internal control	0.784	<0.001
Sexual satisfaction	0.777	<0.001
Sexual self-esteem	0.777	<0.001
Sexual anxiety	0.775	<0.001
Sexual awareness	0.745	<0.001
Sexual management	0.707	<0.001
Sexual preoccupation	0.658	<0.001
Sexual monitoring	0.548	<0.001
Fear of sex	0.368	<0.001
Sexual optimism	0.124	0.079
Sexual assertiveness	0.098	0.167

**Figure 1:**
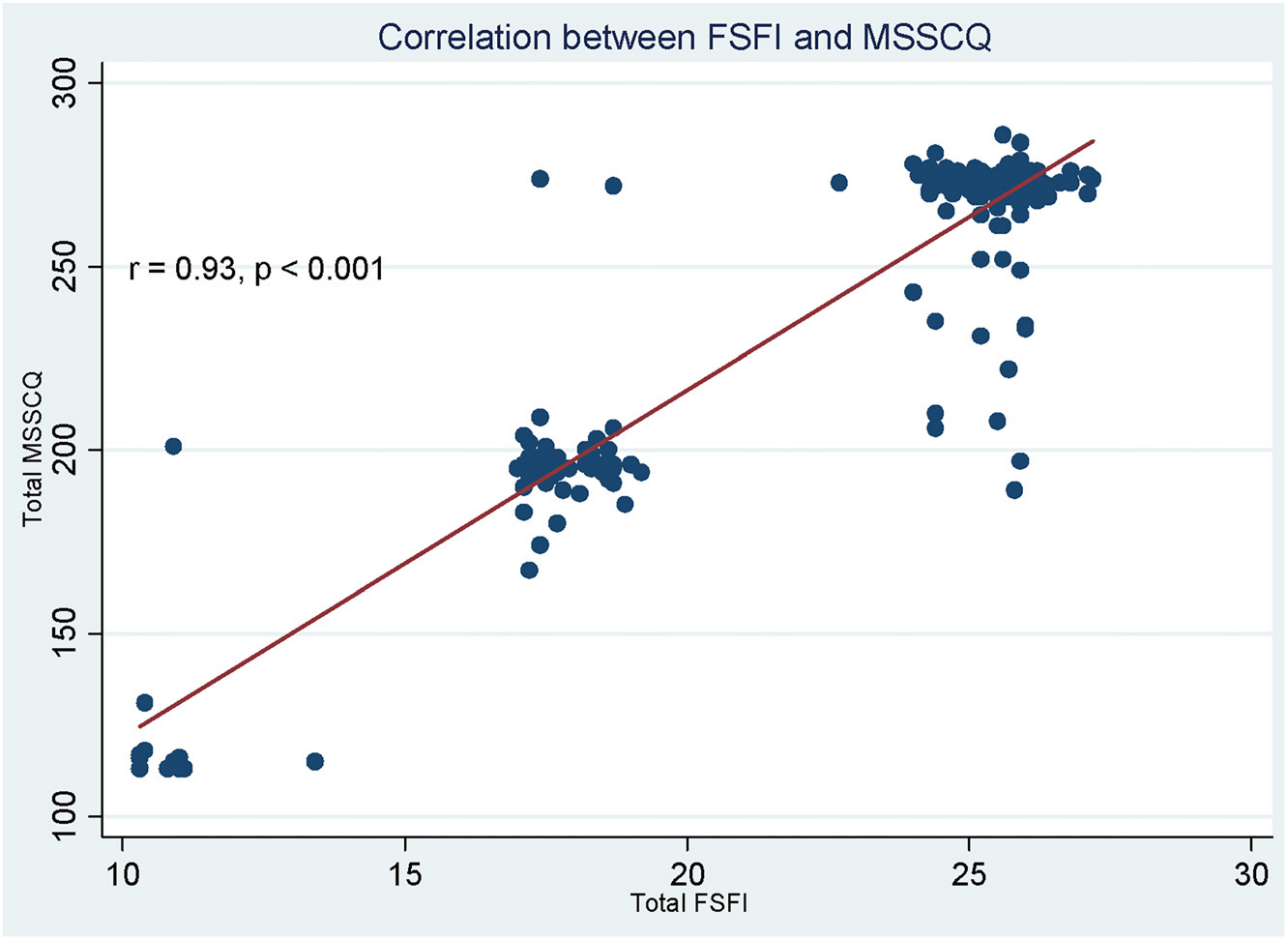
Correlation between total FSFI and total MSSCQ scores in women after hysterectomy.

A strong positive correlation was observed between total FSFI and total MSSCQ scores (r=0.93, p<0.001), as illustrated in Figure 1.

To control for potential confounding variables (age, education, income, and socio-demographic factors), a multivariable linear regression analysis was performed with total FSFI score as the dependent variable. As shown in Table 6, after adjusting for confounders, total MSSCQ score remained a strong and independent predictor of sexual function (B=0.09, 95 % CI: 0.08–0.10, p<0.001). The model explained 87 % of the variance in sexual function (R^2^=0.87).

**Table 6:** Multivariable linear regression for predicting total FSFI score.

Variable	B (unstandardized)	Standard error	95 % CI	p-Value
Total MSSCQ score	0.09	0.005	(0.08 – 0.10)	<0.001
Age	0.02	0.03	(−0.04 – 0.08)	0.452
Income	0.05	0.03	(−0.001 – 0.10)	0.054
Education (ref: Primary)				
Diploma	1.20	0.80	(−0.40 – 2.80)	0.135
University	2.10	0.90	(0.30 – 3.90)	0.022
Husband employed (ref: Unemployed)	1.80	0.70	(0.40 – 3.20)	0.011
Separate bedroom (ref: No)	1.50	0.60	(0.30 – 2.70)	0.014

Table 7 presents the relationships between age, husband’s age, number of pregnancies and children, income, and the two main outcome measures (total FSFI and total MSSCQ). Significant positive correlations were observed between age, husband’s age, number of pregnancies, and number of children (r=0.60 to 0.92, p<0.001), indicating expected associations within age, husband’s age, number of pregnancies and children. Income showed a modest but significant negative correlation with age and reproductive variables (r=−0.16 to −0.36, p<0.05). Notably, total FSFI and total MSSCQ were strongly positively correlated with each other (r=0.93, p<0.001) but negatively correlated with age (r=−0.55 and −0.47, respectively) and number of pregnancies (r=−0.44 to −0.33) and number of children (r=−0.40 to −0.33), suggesting that increasing age and higher reproductive history are associated with lower sexual function and sexual self-concept scores. Income was positively associated with total FSFI and total MSSCQ, albeit with weaker correlations (r=0.26 and 0.23, respectively).

**Table 7:** Pearson correlation coefficients among age, reproductive history, income, and quality of life measures.

Variable	Age	Husband age	No. of pregnancies	No. of children	Income	Total FSFI	Total MSSCQ
Age	1.00						
Husband age	0.92^a^	1.00					
No. of pregnancies	0.69^a^	0.69^a^	1.00				
No. of children	0.60^a^	0.60^a^	0.87^a^	1.00			
Income	−0.34^a^	−0.36^a^	−0.21^a^	−0.16^a^	1.00		
Total FSFI	−0.55^a^	−0.50^a^	−0.44^a^	−0.40^a^	0.26^a^	1.00	
Total MSSCQ	−0.47^a^	−0.44^a^	−0.39^a^	−0.33^a^	0.23^a^	0.93^a^	1.00

^a^p<0.05.

## Discussion

In the present study, 200 women who had undergone hysterectomy were examined. The findings indicated that higher levels of education, the husband’s occupation, ethnicity, frequency of monthly sexual intercourse and having a private bedroom were positively associated with sexual function and sexual self-concept. In contrast, no statistically significant association was observed between housing status or religion and sexual function or sexual self-concept. Furthermore, the study revealed a strong positive correlation between sexual function scores and sexual self-concept scores.

Hysterectomy can be a challenging experience for women. The uterus is a highly important part of the body, and in addition to its role in reproduction, it is also associated with femininity and women’s sexual desires [17].

A study conducted by McCool-Myers et al. demonstrated that multiple factors including poor physical and psychological health, poor sexual health and functioning of the partner, the partner’s unemployment or low educational attainment, stress, history of abortion, menopause, genitourinary problems, female genital mutilation, dissatisfaction with the relationship, history of sexual abuse, and religiosity, were associated with female sexual dysfunction [18]. Similarly, Pasha et al. reported that sexual dysfunction was more prevalent among infertile men who engaged in sexual intercourse less frequently [19]. These findings are consistent with the results of the present study.

In the study by Mohammadi Nik et al. women’s educational level and employment status were significantly associated with a more positive sexual self-concept [20]. Likewise, research by Safshekan et al. revealed that sexual self-esteem scores increased with higher levels of education, women’s employment, longer marital duration, and a positive body image [21]. Since sexual self-esteem is considered one of the components of sexual self-concept, the results of these studies can also be regarded as consistent with the findings of the present research.

In the present study, women’s sexual self-concept significantly increased with higher educational levels of both the women and their spouses, which is consistent with the findings of Jamali et al. [22]. It appears that educated individuals are more engaged in reading and reflection; therefore, they may be more capable of seeking solutions to their problems, leading to greater self-esteem and, consequently, improved sexual satisfaction [22].

Bani et al. reported that the husband’s age and educational level were predictors of a positive sexual self-concept, whereas the woman’s age and the length of premarital acquaintance were predictors of a negative sexual self-concept [23]. The findings of the present study are aligned with theirs regarding the negative correlation between women’s age and sexual self-concept. However, the results diverge concerning the husband’s age, which may be explained by the particular physical, psychological, and social characteristics of the women who have undergone hysterectomy in this study, whose husbands were generally older.

A study conducted by Dedden et al. demonstrated that hysterectomy is not associated with significant changes in sexual function, and hysterectomy with ovarian preservation may even be linked to improvements in certain domains of sexual function, such as lubrication and orgasm [4].

According to the findings reported by Ziaei et al. enhancement of the positive dimensions of sexual self-concept (including sexual self-esteem and sexual satisfaction) and reduction of its negative dimensions (such as sexual anxiety) can promote positive sexual functioning in women of reproductive age [24]. Similarly, the study by Riyazi et al. revealed that sexual motivation and sexual satisfaction play important roles in sexual function among infertile women [10]. Atrian et al. also found a positive correlation between sexual self-efficacy and sexual function [25]. Since sexual self-efficacy, sexual motivation, and sexual satisfaction are considered components of sexual self-concept, these findings are consistent with the results of the present study, which demonstrated a positive correlation between sexual function and sexual self-concept. This research may assist hysterectomized women in gaining a better understanding of their sexual function and self-concept, thereby enabling them to take effective steps toward improving their marital and psychological quality of life.

There were some limitations in this study. a) The identified associations between factors such as education, employment, and ethnicity and the observed outcomes must be interpreted with caution. These factors are frequently linked to other unmeasured socio-economic confounders, which could potentially influence the results. b) The study’s cross-sectional design captures data at a single point in time. While this allows for the identification of correlations, it precludes the establishment of causal relationships between the aforementioned factors and sexual function scores. c) Another limitation of this study is the absence of preoperative baseline data and its cross-sectional nature, which makes causal inference impossible. d) The reliance on self-reported questionnaires for sensitive topics like sexual function introduces the potential for biases, such as social desirability bias and recall bias.

Improving the quality of future research in this field requires attention to several key areas. First, improving study design and modifying sampling methods including expanding inclusion criteria to include women from diverse social, economic, and cultural backgrounds could increase the sample size and improve the generalizability of the results. Second, more careful control for confounding factors related to psychological status, marital relationships and clinical conditions could help to more accurately interpret the observed relationships.

Analytically, it is also recommended that future research use more advanced statistical methods, such as structural equation modeling, to examine the multivariate and complex relationships between sexual self-concept, sexual function, and mediating factors. In addition, subgroup analyses if sufficiently powered could allow for the identification of potential differences across demographic groups.

## Conclusions

This study demonstrated a significant positive correlation between sexual self-concept and sexual function among women who undergone hysterectomy. Preliminary evidence for future longitudinal or interventional sexual research can provide a basis for intervention strategies and evaluate the effectiveness of targeted educational and psychological programs in improving these dimensions.
